# Empyema necessitans with osteomyelitis of fifth rib due to *Nocardia farcinica*: a case report

**DOI:** 10.1186/s12879-021-06452-6

**Published:** 2021-08-03

**Authors:** Swapnil Tripathi, Durga Shankar Meena, Amit Kumar Rohila, Neetha T.R., Vidhi Jain, Deepak Kumar, Taruna Yadav, Mahendra Kumar Garg

**Affiliations:** 1 Department of Medicine, All India Institute of Medical Sciences, Jodhpur, Rajasthan 342005 India; 2 Department of Microbiology, All India Institute of Medical Sciences, Jodhpur, 342005 India; 3 Department of Diagnostic and Interventional Radiology, All India Institute of Medical Sciences, Jodhpur, 342005 India

**Keywords:** Empyema necessitans, Nocardia, Osteomyelitis, Immunocompetent, Case report

## Abstract

**Background:**

Empyema necessitans is a rare pulmonary condition described as the presence of pus in the pleural cavity with insidious extension into the surrounding soft tissue. The common microbial aetiology of empyema necessitans is tuberculosis. Nocardiosis a cause of empyema necessitans is rarely described in the literature. We herein present a case of an 80-year-old male with empyema necessitans with osteomyelitis of rib caused by *Nocardia farcinica*.

**Case presentation:**

An 80-year-old male presented with complaints of soft swelling on the left lower posterior chest wall associated with dry cough and breathlessness on exertion. Computed Tomography (CT) thorax demonstrated empyema necessitans with features of left fifth rib osteomyelitis. Radiological guided aspiration of the chest wall collection revealed *Nocardia species* and surgical drainage of abscess was performed. Matrix-assisted laser desorption/ionization time-of-flight-mass spectrometry (MALDI-TOF-MS) identified the isolate as *Nocardia farcinica*. He was treated with three-drug regimen: Trimethoprim-sulfamethoxazole, amikacin and ceftriaxone for 2 weeks. After showing improvement patient was discharged and advised to take oral Trimethoprim-sulfamethoxazole for the next 6 months with periodic follow-up.

**Conclusions:**

As our case demonstrates, the possibility of invasive Nocardiosis should not be overlooked even in immunocompetent patients. Clinicians should aware of this rare entity while treating patients with empyema necessitans.

## Background

Empyema necessitans (EN) is a complication of untreated empyema thoracis or intraparenchymal pus collection which decompresses through the weak subcutaneous tissue in the chest wall [1]. It is characterized by the communication of the pleural cavity to the skin or soft tissue leading to fistula formation and chest wall swelling respectively. Incidence of empyema necessitans had drastically reduced as compared to the pre antibiotics era with *Mycobacterium tuberculosis* still remaining the commonest cause (nearly 70%) followed by Methicillin resistant *Staphylococcus aureus* (MRSA) [2, 3]. Bacterial causes of EN are rare particularly Nocardia, with only 3 cases reported in the literature so far [4–6]. Moreover, Nocardia osteomyelitis involving sites other than spine is also rare with only a handful of cases reported to date and only one with rib osteomyelitis [7–10]. Here we report a case of an 80-year-old male presented with empyema necessitans and fifth rib osteomyelitis who has no known risk factors for invasive Nocardiosis.

## Case presentation

An 81-year-old male presented to our hospital with complaints of dry cough and progressive shortness of breath for the last 3 months. He also had low grade fever for the last 15 days. There was no history of orthopnea, weight loss, pain abdomen or pedal edema. Significant past history included hypertension for the last 10 years and coronary artery disease and percutaneous angioplasty performed 5 years back. He was on aspirin, beta blockers and ramipril. On examination, there were stony dullness with decreased breath sounds in left infra scapular and axillary region. Chest roentgenogram suggestive of left side pleural effusion and aspiration showed pus in pleural cavity. Laboratory evaluation of collection was exudative in nature and was negative for *Mycobacterium tuberculosis* on ZN staining (20% H_2_SO_4_) and gene-Xpert. BacT/Alert liquid culture for *Mycobacterium tuberculosis* was also negative. Patient was treated with empirical antibiotics (amoxicillin-clavulanate 1.2 g intravenous 3 times a day) with mild symptomatic improvement and was discharged subsequently. However, he was re-admitted after 20 days with complaints of swelling on back (left posterior chest wall). The development of swelling was within the last 20 days and it did not bother the patient until recently when it became painful.

**Fig. 1:**
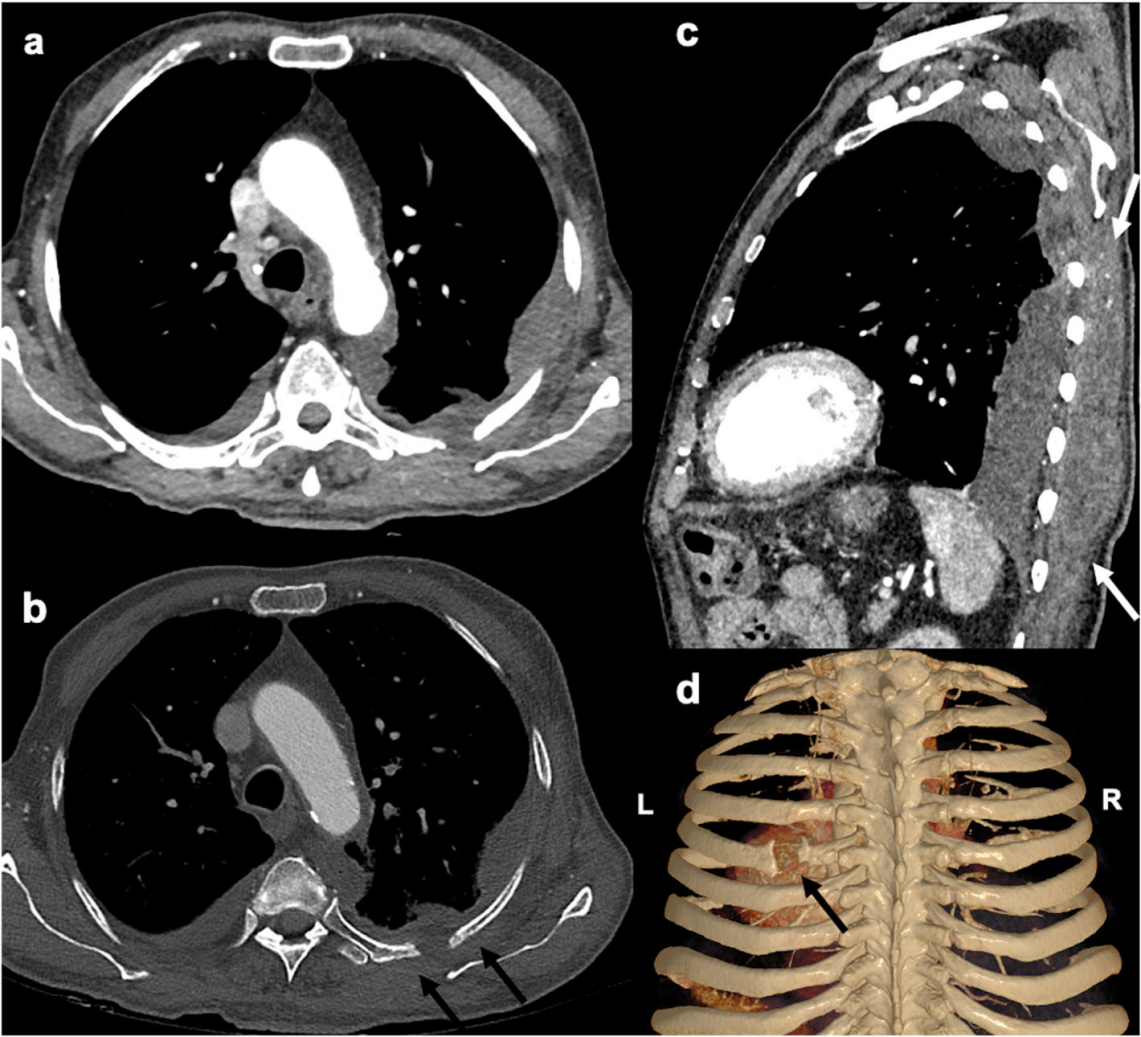
Contrast enhanced computed tomography (CECT) scan images showing rib osteomyelitis with empyema necessitans. (**a**, **b**) Axial images in (**a**) mediastinal, (**b**) bone windows showing left sided loculated pleural effusion with mild right sided pleural effusion. Black arrows in **b** show the defect in the involved rib with sclerotic changes in the remaining rib bone marrow. **c** Sagittal image shows the pleural fluid extending into the chest wall till subcutaneous tissues plane (white arrows). **d** Volume rendered image shows the rib defect with associated periosteal reaction (black arrow)

At presentation, his vitals were stable except tachypnea (respiratory rate of 24/min). He was afebrile and maintaining oxygen saturation of 93% on ambient air. Physical examination revealed pallor and a soft, fluctuant, non-tender swelling of size 12 cm X 8 cm on the left lower posterior chest wall. On auscultation, air entry was reduced on the left infra scapular and infra-axillary region with decreased vocal fremitus. Rest systemic examination was unremarkable. Blood investigations showed anemia (hemoglobin 8.9 g/dl), raised erythrocyte sedimentation rate (58 mm), elevated highly sensitive C-reactive protein (91.5 mg/dl, normal < 1 mg/dl), and mildly raised procalcitonin (0.80 ng/ml, normal < .02 ng/ml). Chest roentgenogram again suggestive of left-sided pleural effusion. His aerobic and anaerobic blood cultures were sterile. On further investigation, Computed tomography of the chest (Fig. 1 a, b, c, d) revealed left-sided moderate pleural effusion with a posterior chest wall collection of size 12.8 × 8.2 × 3.2 cm below the latissimus dorsi with the evidence of fifth rib osteomyelitis. Since the disease was progressive despite taking antibiotics, we kept the differentials of tuberculosis and malignancy however we did not have any evidence of malignancy on radioimaging. In addition, gene Xpert was also negative which ruled out tuberculosis.

**Fig. 2:**
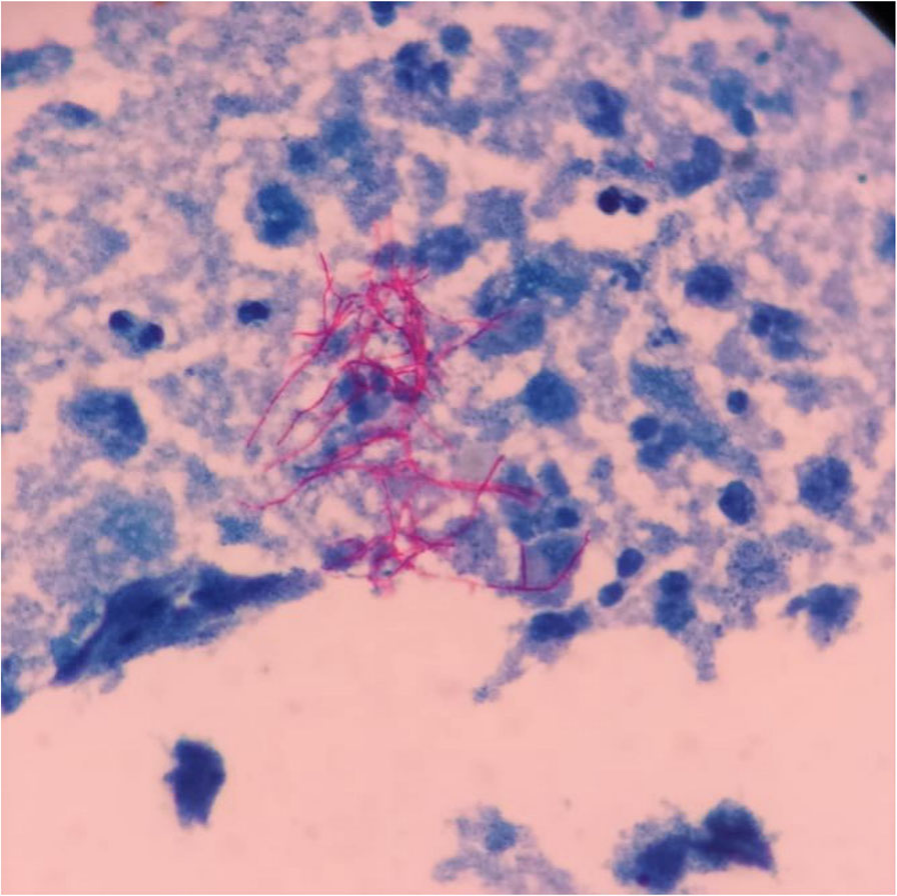
Direct microscopic appearance of aspirated pus after ZN staining (Modified Kinyoun technique with 1% sulphuric acid) showing thin, slender, acid-fast, beaded, branched bacilli against a background of pus cells, suggestive of Nocardia Spp

**Fig. 3:**
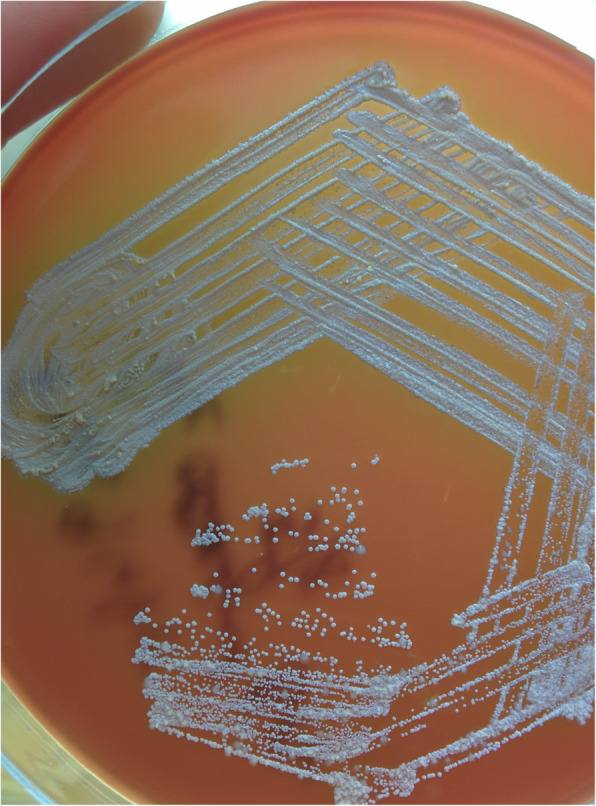
Aerobic culture on 5% Sheep blood agar showing small dry white stony colonies of Nocardia Spp. on day 8 of incubation at 37 °C

Ultrasound guided aspiration of the posterior chest wall collection was performed. Gram staining and modified Ziehl-Neelsen (ZN) staining (1% H_2_SO_4_, Modified Kinyoun method) of the aspirated pus revealed plenty of pus cells and numerous gram positive, acid fast thin slender, beaded, branching bacilli, suggestive of *Nocardia Spp* (Fig. 2). Routine aerobic culture on 5% sheep blood agar and Mac Conkey’s agar showed the growth of small whitish to brown, dry, stony hard colonies after 5–6 days of incubation at 37 °C. The isolate was identified as *Nocardia Spp* based on colony morphology and characteristic staining (Fig. 3).

The patient was managed with therapeutic aspiration of chest wall collection and chest tube was inserted for empyema. Three-drug regimen consisting of oral trimethoprim-sulphamethoxazole (600 + 3000 mg/day in two divided doses), intravenous amikacin (900 mg once daily) and intravenous ceftriaxone (1 g twice a day) was started. MALDI-TOF confirmed the species as *Nocardia farcinica*. The isolate was susceptible to most antibiotics (ceftriaxone, imipenem, amikacin, cotrimoxazole, minocycline and linezolid) except ampicillin. On day 14 of hospitalization, patient showed significant improvement and shifted to oral trimethoprim-sulphamethoxazole which was scheduled to continue for the next 6 months.

## Discussion and conclusions

Empyema necessitans was predominantly a disease of the pre-antibiotics era with only a few cases being reported after the availability of highly effective antibiotics. It is a rare complication of parapneumonic effusion or untreated empyema thoracis which extends through the parietal pleura into the subcutaneous tissue and skin. The most common location for empyema necessitans is the anterior chest wall, the other sites are back, diaphragm, mediastinum, and esophagus. Apart from *Mycobacterium Spp*, other common microbial etiology of empyema necessitans include *Staphylococcus aureus, Streptococcus Spp, Fusobacterium Spp, and Proteus Spp* [2].

Nocardia is a non-fastidious aerobic, gram-positive, acid-fast filamentous, branched bacillus [11]. Out of more than 85 species identified, nearly one-third of them cause infection in humans [12]. It is considered an opportunistic pathogen with around two-third of the cases occurring in immunocompromised individuals. Common species causing human infections include *N. asteroidis complex, N brasiliensis, N. abscessus,* and *N cyriacigeorgica* [12]. Pulmonary disease is the most common manifestation of Nocardiosis, with other organs involves are the central nervous system (CNS), skin, and soft tissue. The pulmonary manifestation of Nocardiosis are acute or chronic pneumonia, lung abscess, pleural effusion, and pulmonary nodule. Our patient was immunocompetent despite that he developed empyema necessitans related to Nocardia. On literature search, only 3 cases of empyema necessitans caused by *Nocardia Spp* have been reported [4–6]. The first report of Nocardiosis causing EN was published by Cadena et al. in 2008 [4]. In their report, Nocardia *asteroids* was the organism responsible for EM in a 59-year-old immunocompetent male. Another report by Severo et al. described a case of a 59-year-old male with history of lung transplant (alpha-1 antitrypsin deficiency) presenting as empyema necessitants caused by *Nocardia Nova* [6]. In their case, infection was spread to the mediastinum and also caused pericarditis. Among these previously reported cases, there was no description of rib osteomyelitis. Bishara et al. performed a review of 106 cases of rib osteomyelitis and described the various etiologies [13]. Mycobacterial and bacterial infections accounted for 47 cases each followed by fungal infections (11 cases). one case was caused by *Entamoeba histolytica* [13]. Bone disease with osteomyelitis is unusual in Nocardiosis which rarely affects small and flat bones. Mode of transmission is predominantly hematogenous that explains its involvement of long bones which are more vascular and metabolically active [14]. The other route of transmission are direct inoculation by trauma or spread from adjacent tissue which was the case with our patient. He had pulmonary nodules and rib osteomyelitis along with EN which pointed the initial diagnosis towards malignancy. The other possibility was tuberculosis especially in the setting of developing countries [15]. Clinician should aware of invasive nature of Nocardiosis which can mimic malignancy, tuberculosis and sometimes an invasive fungal infection.

Diagnosis of nocardiosis can be made using direct microscopy and culture. Using modified ZN staining (using 1% sulphuric acid), *Nocardia Species* can be seen as filamentous, branching and beaded acid fast bacilli. Further species identification may be performed by using polymerase chain reaction and 16S rDNA sequencing. Recently MALDI-TOF has been used for species identification which is a rapid, accurate and cost-effective alternative of gene sequencing methods. Empirical antibiotic coverage with at least 2 antimicrobials should be given due to variable resistance related to different Nocardia species. The cornerstone of the treatment of Nocardiosis is Trimethoprim-Sulfamethoxazole at the dose of 5-10 mg/kg/day of Trimethoprim component. Alternative treatment includes third-generation cephalosporins, carbapenems, amikacin and linezolid [16]. The duration of therapy depends upon clinical syndrome and immune status of patient. Pulmonary disease in immunocompetent patients requires at least 6 months of therapy while disseminated and CNS disease requires 12 months treatment [11]. Surgical drainage of abscess can augment the disease recovery. For Nocardia osteomyelitis surgical debridement is crucial with at least 4 months of antibiotics. Though one report showed good recovery with conservative treatment [8].

Tuberculosis is still the commonest cause of empyema necessitans in developing countries. However, the possibility of Nocardiosis should be considered by the clinicians wherever tuberculosis has been ruled out. Microbiologist and clinician synchronization is crucial for early diagnosis of Nocardia. The current case illustrates the importance of targeted sample collection and high-quality microscopy providing the earliest diagnostic evidence of an uncommon causative agent within a few hours. Such timely diagnosis is important especially those with underlying immunocompromised state to prevent significant morbidity and mortality in these patients.

## Data Availability

The datasets used and/or analysed during the current study available from the corresponding author on reasonable request.

## Abbreviations

EN: Empyema Necessitans
CT: Computed Tomography
MRSA: Methicillin resistant *Staphylococcus aureus*
ZN staining: Ziehl–Neelsen staining
Spp: Species
CNS: Central Nervous System
